# Community-driven governance of FAIRness assessment: an open issue, an open discussion

**DOI:** 10.12688/openreseurope.15364.1

**Published:** 2022-12-23

**Authors:** Mark D. Wilkinson, Susanna-Assunta Sansone, Eva Méndez, Romain David, Richard Dennis, David Hecker, Mari Kleemola, Carlo Lacagnina, Anastasija Nikiforova, Leyla Jael Castro

**Affiliations:** 1 EOSC Task Force on FAIR Metrics and Data Quality, EOSC, Brussels, Belgium; 2 Departamento de Biotecnología-Biología Vegetal, Escuela Técnica Superior de Ingeniería Agronómica, Alimentaria y de Biosistemas, Centro de Biotecnología y Genómica de Plantas. Universidad Politécnica de Madrid (UPM) - Instituto Nacional de Investigación y Tecnología Agraria y Alimentaria-CSIC (INIA-CSIC), Madrid, Spain; 3 Department of Engineering Science, Oxford e-Research Centre, The University of Oxford, Oxford, UK; 4 Library and Information Science Department, Universidad Carlos III de Madrid, Madrid, Spain; 5 European Research Infrastructure on Highly Pathogenic Agents (ERINHA AISBL), Brussels, Belgium; 6 Novo Nordisk Foundation Center for Stem Cell Medicine – reNEW, University of Copenhagen, Copenhagen, Denmark; 7 Research Data Management, German Aerospace Center (DLR), Cologne, Germany; 8 Finnish Social Science Data Archive and CESSDA ERIC, Tampere University, Tampere, Finland; 9 Barcelona Supercomputing Center, Barcelona, Spain; 10 Institute of Computer Science, The University of Tartu, Tartu, Estonia; 11 Semantic Technologies team, ZB MED Information Centre for Life Sciences, Cologne, Germany

**Keywords:** FAIR, FAIR evaluators, FAIRness assessment, Governance

## Abstract

Although FAIR Research Data Principles are targeted at and implemented by different communities, research disciplines, and research stakeholders (data stewards, curators, etc.), there is no conclusive way to determine the level of FAIRness intended or required to make research artefacts (including, but not limited to, research data) Findable, Accessible, Interoperable, and Reusable. The FAIR Principles cover all types of digital objects, metadata, and infrastructures. However, they focus their narrative on data features that support their reusability. FAIR defines principles, not standards, and therefore they do not propose a mechanism to achieve the behaviours they describe in an attempt to be technology/implementation neutral. A range of FAIR assessment metrics and tools have been designed that measure FAIRness. Unfortunately, the same digital objects assessed by different tools often exhibit widely different outcomes because of these independent interpretations of FAIR. This results in confusion among the publishers, the funders, and the users of digital research objects. Moreover, in the absence of a standard and transparent definition of what constitutes FAIR behaviours, there is a temptation to define existing approaches as being FAIR-compliant rather than having FAIR define the expected behaviours. This whitepaper identifies three high-level stakeholder categories -FAIR decision and policymakers, FAIR custodians, and FAIR practitioners - and provides examples outlining specific stakeholders' (hypothetical but anticipated) needs. It also examines possible models for governance based on the existing peer efforts, standardisation bodies, and other ways to acknowledge specifications and potential benefits. This whitepaper can serve as a starting point to foster an open discussion around FAIRness governance and the mechanism(s) that could be used to implement it, to be trusted, broadly representative, appropriately scoped, and sustainable. We invite engagement in this conversation in an open Google Group fair-assessment-governance@googlegroups.com.

## Disclaimer

The views expressed in this article are those of the author(s). Publication in Open Research Europe does not imply endorsement of the European Commission.

## 1. Introduction

The publication of the FAIR Principles in 2016 heralded a new era for data-driven research, improving the focus on the Findability, Accessibility, Interoperability, and Reusability of research outputs ^1^. Multiple communities in different domains are now implementing these Principles and actively evolving this work worldwide. Recent initiatives include increasing the development of tools to assist with achieving FAIRness of research data and adapting the FAIR Principles to other digital research objects such as software, workflows, training material, and tools that assess the degree of FAIRness achieved. There are also discussions around expanding the FAIR Principles, or their interpretation, to include features of digital objects beyond reusability, including popularity ^2^, data quality ^3^, or reproducibility ^4^. Different research funders in other countries require researchers to make underlying research data "as open as possible and as closed as necessary" and follow the FAIR Principles. Likewise, the current work to reform the research assessment system ^5^ includes data sharing as part of the qualitative research evaluation. Still, there is no agreement on the quality of research data sharing, "FAIRness," and how to measure it.

FAIR comprises principles, not standards with which one is expected to comply. Thus, research performing organisations, researchers, and managers need to ensure that data and all other Digital Objects are FAIR-compliant research outputs without a body or committee that clarifies what FAIRness "means" and how FAIRness validation tools can support it.

From within this milieu, the topic of FAIRness governance is arising. Governance here is understood as a community-driven and agreed way of providing reliable, trusted assistance and tooling to improve FAIRness and should not be interpreted as an attempt to impose judgment. Improved FAIRness is the goal of many major international initiatives, including the European Open Science Cloud (EOSC) and funding agencies at the national and international levels.

This whitepaper evolved from discussion at the 2021 IEEE eScience workshop, FAIReScience, and individual meetings among the authors. All of them are involved in the FAIR community as original authors of the Principles, FAIR project leaders, advocates, implementers, technology developers, trainers, advisors, and stewards. The draft whitepaper was then reviewed and revised by the members of the EOSC Task Force on FAIR Metrics and Data Quality as part of their charter requirement to examine FAIR evaluation and governance issues. Task Force members were invited to become co-authors. The EOSC Board then reviewed this final revision before publication. This document aims to create a clear argument for the need for a governance model around a standard definition of FAIRness, demonstrate how stakeholders will benefit from it, and explore existing governance models from peer internet projects to understand better the decisions that need to be made. The authors aim to foster an open debate with the community around FAIRness governance and the mechanism(s) that could be used to implement it to be trusted, broadly representative, appropriately scoped, and sustainable. It begins by detailing the needs based on use cases relevant to various stakeholders. It then examines possible models for governance, reviewing existing and peer efforts in this public domain.

## 2. Rationale

The FAIR Principles lay out a set of features that a digital object should exhibit to optimise its ability to be discovered and correctly reused, focusing on fully mechanised reuse. The FAIR Principles are meant to cover all sorts of digital objects. However, they focus their narrative on data features that support their reusability. The Principles do not propose a mechanism to achieve the behaviours they describe in an attempt to be technology/implementation-agnostic. This flexibility has led to many interpretations of what it means to "be FAIR," with early claims from essential resources that they already "were FAIR. “Given that FAIR was intended to spur a revolution in research scholarship, and given that the degree of interoperability achieved by the *status quo* is severely lacking, claims to pre-existing or newly acquired FAIRness needed to be objectively examined.

In the last few years, several activities have focused on developing FAIR metrics/maturity indicators and tests, measurable features of a digital object that correspond to its compliance with a specific FAIR Principle, and tools to fully or semi-automatically implement these tests. Unfortunately, these tools often have a different interpretation of the original intent of each Principle and, therefore, of what needs to be tested and how. For example, various tools follow different workflows for harvesting the metadata about a digital object upon which they will execute their tests, and may not find all possibilities, or worse, may see unrelated metadata because of following a path that the digital object provider is using for another purpose. As a result, digital objects evaluated by different tools often exhibit widely different outcomes, which confuses both the publishers and consumers of digital objects, hindering them from applying corrective actions. Therefore, these evaluation initiatives need guidance to ensure that their FAIRness assessments are (a) within the scope of the FAIR Principles and (b) consider the full breadth of valid technical options to enact the required metadata gathering and testing behaviours. Given the increasing number of Web standards compliant with FAIR Principles, such as the emergence of RO-Crates ^6^ as a mechanism to publish data-provider-sourced domain metadata, it is essential to ensure that "objectively compliant" resources are not judged "unfairly" due to their selection of one technology or standard versus another.

Moreover, FAIR is an impetus toward the proper management of digital objects, which is essential for the greater good. Therefore, FAIRness must not be used or perceived as an instrument to judge or punish. The community is genuinely concerned that mechanisms to evaluate FAIRness can be misused and misinterpreted, especially when these become a decision-making instrument in funding scenarios. Thus, governance of FAIRness assessment will better serve the community if it is focused on assisting and guiding stakeholders to reach a reasonable level of FAIRness or to assist in the interpretation of the facets of FAIRness that are achieved by a given digital object before deciding to use it (or not), versus using assessments as a path to judgment. Thus, beyond providing rapid pass/fail outputs for any given assessment metric, consideration should be given to the supportive advice given by the evaluation systems in response to various kinds of failure, and this advice given would benefit from being harmonised between all evaluation systems to avoid divergence in the community.

This work notes that there have been movements to expand the FAIR Principles to include features of digital objects beyond reusability, including popularity and data quality. While these latter features are undoubtedly essential and of interest to many stakeholders, they do not directly fall within the scope of the FAIR Principles. They are thus not included in this discussion. This careful scoping of FAIRness around the original Principles is even more important in light of the knowledge that the FAIR Principles anticipated, from the outset, that they would need to be fine-tuned and interpreted for individual research communities - with the final FAIR Principle (R1.3) stating that a FAIR digital object should follow community standards. Thus, there will be, intentionally, an expansion of the expected FAIRness behaviours over time, as individual communities circumscribe the FAIR expectations within their domains. Ensuring high quality, reliable, and appropriately focused community-driven assessments will also require a governance process that is transparent and broadly inclusive, and this has been a focus of the EOSC Task Force on FAIR Metrics and Data Quality. Finally, there is also an active movement around the adaptation of the FAIR Principles to digital objects other than data, e.g., software and workflows, with few tools to evaluate their FAIRness. A similar trend could be expected, i.e., variety in interpreting the FAIR Principles as they are applied to these non-data objects. Similar to the data case, this would lead to incoherent FAIR assessments between employed [tools/tests] in the absence of FAIRness governance.

Nevertheless, and importantly, this whitepaper does not suggest that the FAIR Principles themselves require governance. The FAIR Principles represent a milestone in the evolution of scholarly data reuse and likely should remain untouched as written; specialised domains should extend those Principles autonomously to make them more relevant to their community. Those extensions, however, should be associated with transparent and objective assessment tools, which we suggest should be subject to review by the proposed governance process and should evolve together with the extensions and interpretations and implementations of the principles over time.

Box 1 below summarises the scope of this whitepaper and its critical objectives for defining a governance model, which is (i) motivated by the need to have understandable and trustworthy claims of FAIRness, and (ii) focused on helping evaluation tools and services to deliver transparent and consistent results. The latter means what should be tested, how it aligns with metrics and maturity indicators, and how FAIRness levels can be presented qualitative or quantitatively.

**Box 1. FAIRness governance model: key objectives and indicators of success**

How will we know when we have identified an effective governance model for the FAIR assessment? We think it will have the following features:

1. FAIRness will be objectively examined transparently and consistently:
  The results of different tools and services that assist and evaluate levels of FAIRness are compatible and cohesive within each domain/community and, as much as possible, between communities.
2. FAIRness needs to be universally understood and trusted:
  ◦ Both producers and users of digital research objects have confidence in the results of these tools, processes, and services;
  ◦ Producers are assisted in their attempts to improve their level of FAIRness.
3. FAIRness needs to be tuned to all domain-specific needs and adapted to a diverse range of digital objects:
  ◦ Communities have a process in place to collect requirements from their members;
  ◦ All stakeholders ensure transparency, consistency, understandability, broad acceptance, and trust.

## 3. Stakeholders groups and their use cases

Ten stakeholders are identified below that can benefit from a FAIRness governance model. These are split into three main groups as follows:

- The first group (A), **FAIR practitioners**, corresponds to those directly working on research, including individual researchers and domain-specific research communities, whose responsibility it is to apply the FAIR Principles to their work - both as consumers and producers of data - and utilise services that assist them in achieving this.
- The second group (B), **FAIR custodians**, corresponds to stakeholders that will support FAIR in practice, also via research on FAIR itself, recommendations for FAIR adopters, or provision of tools making it easier for researchers to produce FAIR research and for those in the first group to assess FAIRness requirements. In this group, there are FAIR researchers, i.e., researchers whose field of research are the FAIR Principles and elements around them, and FAIR stewards, i.e., data or any other digital object stewards supporting the FAIR Principles.
- The third group (C), the **FAIR decision and policymakers**, encompasses stakeholders that will require access to FAIRification plans, i.e., how the FAIR Principles will be supported and achieved, for different digital objects involved in a research process but is not in charge of FAIRifying those digital objects themselves. This group includes funding agencies, governments, and publishers.

The list of stakeholders mentioned in the sections below is not intended to be comprehensive but just examples. This list of stakeholders is largely alighted with those identified by the Research Data Alliance Sharing Rewards and Credit Interest Group (RDA-SHARC-IG) ^7^.

### 3.1. Group A: FAIR practitioners

***3.1.1. Researchers and research-performing organisations (domain-agnostic).*** For this document, researchers are any individual or team that performs research, produces research outputs, and applies for grants to conduct that research. This includes research professionals from all or any field or discipline.

**Use-case**: A researcher has completed a project and is preparing to engage in the final stages of their data stewardship plan. This requires them to publish their data following the FAIR Principles. They have a variety of options, including (i) self-publication on the Web; (ii) publication in their domain-specific institutional repository; (iii) publication in one of the several generic repositories. How can the researcher find the best option for his data that is also accepted by his funding body, especially if the option incurs costs (e.g., self-publication, large datasets)? Because the FAIRness governance body has endorsed and vetted a set of community accepted metrics and tests, the researcher can compare the options easily by employing these tests using trusted and objective assessment tools. The test reports can then justify the decision, including possible costs (monetary, human resources), to their funding agency.

***3.1.2. Research software engineers.*** Research Software Engineers (RSE) are well-recognised stakeholders. They include those professionals who combine software expertise with an understanding of research, such as software developers supporting research development. Researchers are developing software to collect, process, analyse, host, publish, and preserve data in this context.

**Use-case**: A research developer in the Botanical field wants to assess the software's FAIRness (either open-source-code or not) and data, e.g., datasets or ontologies, used for their research. They register and richly describe these resources in the FAIR-4-Plants registry (the one recommended by their community), using FAIR-relevant descriptors such as terms of access, information on the use of (meta) standards, and identifier schema. The engineer can start the self-assessment of these resources using any assessment tool recognised by the FAIRness governance body. They select the one preferred within their community, knowing that, regardless of what other FAIRness tools may be used by their funders or the publishers to whom they submit their research results, they can trust that the FAIRness assessments will be compatible and will accurately reflect their FAIRification efforts.

***3.1.3. Domain-specific research communities.*** Different expert domains encounter dramatically different datasets – in terms of complexity, quality, secrecy, and volume (to name only a few). Thus, various domains will look at the FAIR Principles, and their assessment, through the lens of the kinds of data they collect, and the metadata requirements surrounding their domain-specific datasets.

**Use-case:** A group of researchers in the area of clinical trials creates a GO FAIR Implementation Network to identify and develop FAIR standards for clinical trial data. They agree that a pre-registration document is an element of metadata that should be required for any clinical trial. They define a novel FAIR metric specific to their community that says the metadata record for a clinical trial must contain a reference to a pre-registration document or an equivalent Web landing page. The pre-registration must be retrievable via HTTP(S). They then design the test for this Metric, wherein they decide that a specific predicate should point to this landing page/document. However, they cannot find a suitable predicate in any existing ontology, so they "mint" a predicate "has-pre-registration" themselves. Finally, they submit the Metric, and its test, to the FAIRness Governance body. The Metric is approved; however, the FAIR experts in the governance body suggest that minting novel predicates is not an optimally FAIR activity. The FAIR experts suggest that a more general predicate, such as "conforms to" or "guided by" or "regulated by," would be more appropriate and that these predicates exist in widely used ontologies. In a potentially iterative process, the test is re-written and re-evaluated and eventually became the recommended test for the clinical trials "community" ("community" is defined as those who self-identify as stakeholders in clinical trial data reusability). FAIRness governance experts have vetted the community-based Metric and, therefore, will be trusted by (for example) their funding agencies. Moreover, the FAIRness governance experts, having a broader view of how various communities represent these kinds of problems, can suggest that they follow a model likely to be used by other communities that have processes guided by protocols/methodologies. Thus, not only the clinical trials community but other communities and stakeholders benefit from the activities of the governance body.

### 3.2. Group B: FAIR custodians

***3.2.1 FAIR support stewards & trainers.*** FAIR Stewards and Trainers support community-specific researchers to create FAIR digital objects either by design or FAIRification processes. FAIR stewards facilitate communication between data owners and FAIR experts, collecting and curating the FAIRification challenges passed from owner to expert and curating and interpreting the suggested solution. A FAIR steward would also be responsible for advising on developing, implementing, and monitoring an RDM policy, including support of FAIR data and Open Science principles ^8^.

**Use-case:** The role of FAIR Stewards and Trainers is to guide and assist data producers in making “good choices” regarding technologies and standards to be used to achieve compliance with the FAIR Principles. A first step in performing the role of a Steward or Trainer is increasing FAIR literacy ^9^ for both data producers and FAIR implementers. In this role, there may be a scenario where a data producer approaches their FAIR Steward excited to have found an ontology that contains the precise concept they wish to describe in their data and asks that it be added to their metadata. The FAIR Steward runs a series of FAIRness assessments on the ontology. They find that the ontology does not meet expected FAIR standards (e.g., the terms do not resolve or that there is no usage license). The FAIR Steward then contacts the FAIRness governance body to ask for advice. The FAIRness governance body engages with the ontology authors. It gives them advice on how to improve their resources, making them more FAIR. The FAIR Steward can confidently utilise this metadata with the assurance that it is at a sufficient level of FAIRness. Optimally, this process would take a few days to give timely feedback to the data producer. However, there may be cases where the request necessitates more extensive changes/development.

***3.2.2 FAIR repositories.*** Organisations or infrastructures support researchers in opening, sharing, or preserving FAIR digital objects.

**Use-case:** The repository needs to show its host organisation and funders that their data holdings and practices are FAIR. The repository is discipline-specific and has a mission for the long-term preservation of digital objects. They define a new metric specific to their community that says the metadata record must contain certain elements required for long-term preservation and a test for this Metric. Like the use case in 3.1.3, they submit the Metric and its test to the FAIRness Governance body for feedback and vetting. Furthermore, since the repository's mission is long-term preservation, their collections include digital objects from a prolonged period, and they wish to keep them FAIR over time. Since that is not a trivial task and requires resources, the repository wonders how long a FAIR score can be valid, are old versions of tests or metrics are still valid, and what is a sufficient level of FAIRness over time. The repository contacts the FAIRness governance body to ask for advice. The FAIR governance experts provide recommendations about the validity of older versions of metrics and tests. They might even be able to recommend a certification body for the repository to consider (see 3.2.5). Based on the feedback and recommendations by the FAIRness Governance body, the repository can improve its practices, add in their website information about their FAIRness and how that has been measured so that the funding agencies (as well as users) can easily see, without running tests themselves, that the repository is enabling FAIR and that the digital objects within the repository are FAIR at a sufficient level.

***3.2.3. FAIR tools developers and operators.*** Software developers support and maintain FAIR deployment tools and infrastructure, including metrics designers and evaluators.

**Use-case**: A discipline-agnostic community working on dataset aggregation has identified four mandatory metadata elements and three recommended ones that data providers should support so that data aggregators can present a coherent summary of aggregated datasets. They have found that the complete set of four mandatory metadata elements is present in any dataset regardless of discipline. In contrast, the three recommended ones are only possible for some domains. They have designed a software-based set of FAIRness tests that explores metadata records for datasets and validates whether the four mandatory elements are present. The three recommended ones are only for those disciplines where they are valid. They have defined the conformance rules, i.e., possible values and cardinality. They have presented this to the FAIRness governance body for endorsement. After accepting several suggestions for edits, the novel set of tests becomes endorsed for use. Funding and administrative bodies can now confidently interpret a dataset provider's success in conforming to this novel metadata standard. They have followed the guidelines provided by the FAIRness governance body, as they want their tool to be recognised and trusted by dataset aggregators.

***3.2.4. FAIR researchers.*** This might include "metadata architects," EOSC researchers, PID implementers, and all those communities interested (from different disciplines, backgrounds, or interests) in developing FAIR principles from a technical implementation point of view.

**Use-case:** A small enterprise has created a device that continuously monitors blood sugar in diabetic patients. The data stream from this device is designed to gather and publish additional contextual metadata that can help the patient's clinician better understand the patient's lifestyle and how this affects their blood sugar throughout the day. The metadata does not follow an existing standard. However, it combines several existing ones with some new elements to better reflect their model and the data they collect. The small enterprise has made significant efforts to ensure that their data stream is FAIR. They include this in their advertising for the product. A large medical devices corporation approaches them with an interest in licensing the product. However, it challenges them on the claim of FAIRness - "prove it"! The small enterprise selects the appropriate set of governance body recommended metrics/tests for streaming biomedical data and generates a report that shows their compliance. Because the governance body has vetted the tests, the corporation is reassured that the claims are valid and licenses the product.

***3.2.5. FAIR certification bodies.*** These bodies have established a set of FAIR compliance standards beyond individual FAIR metric tests - e.g., the minimal subset of indicators, properties, or elements that must be met by a dataset or data collection to become "FAIR certified" by this organisation.

**Use-case:** An organisation offers FAIR certification for tools and data repositories as one of its services - an "I'm FAIR!" badge that can be applied to the website to let its users know that the certification body's tests are compliant with the FAIR Principles and accurately assess the FAIR behaviours promoted by the community they are serving. Certification providers, therefore, must demonstrate that the possession of their badge is a valid indication of conformance with FAIR that their clients will trust. Rather than designing their own set of tests, the certification authority board selects metrics, tests, and testing software that have been recommended by the governance body and uses these in their certification workflow. They display the chosen metrics and tests publicly in their advertising, showing that these tests are governance-body recommended. In addition, they submit their certification evaluation workflow to the governance body to ensure it is entirely FAIR. This ensures that their certification is credible, giving it added value.

### 3.3. Group C. FAIR decision and policymakers

***3.3.1. Funding agencies.*** The funding agencies referred to in this first group of stakeholders are institutions calling for grant applications, assessing the applications, providing financial support, and following up on the development of granted projects.

**Use-case**: A funding agency, either public or private, commonly requires something in return for the investment provided via a grant, e.g., providing results back to the community. To this end, a funding agency would include a data stewardship component in its funding applications. They require that data be published using FAIR formats and "as open as possible and closed as necessary. “A successfully funded project in Rare Diseases submits its final report, claiming that they have made their data FAIR but that it cannot be made public due to its sensitivity. This may be the case where the funding body imposes the relevant restrictions or are imposed by the specificity of the data collected and the object on which they are collected. However, real-world experience also pointed to cases where these requirements are misunderstood (unknowingly or even deliberately) and misinterpreted, e.g., lack of knowledge or competence, resistance to fulfilling considering the complexity of the task and resources (time, human, etc.). To be allocated etc. Needing to assess this claim to FAIRness, when the agencies cannot evaluate it themselves, they provide the project leaders with one or more assessment tools that the FAIRness governance body has endorsed. The project leader runs the tool on their data and submits the report to the agency. The agency can trust the assessment results because the governing body has approved its use.

***3.3.2. Governments.*** Under the “governments," stakeholder group includes government bodies shaping a particular economic area such as agriculture or disease control.

**Use-case**: Depending on, for instance, geographical and economic characteristics, a government might want to favour the development of specific socio-economic outcomes. A tropical country with high incidences of malaria would wish to minimise or eradicate the disease. It would be necessary to understand how the infection propagates, what facilitates the spread, and how it can be prevented, requiring data collection and analysis. The government of this country can define policies to encourage and promote FAIR research efforts around malaria and pass these through the FAIRness governance body to ensure they are properly scoped. In this way, researchers can more easily share results and build upon what others have done simply by following governmental policy. Such policies will influence researchers, research communities, national funding agencies, and research evaluation agencies.

***3.3.3. Publishers.*** In this group are included journal publishers that establish publishing policies, including the publication of research data and other research results, as well as other publishers that might be important in different research fields (e.g., publication platforms, learned societies, etc.).

**Use-case:** Research publishers aim at providing trusted research results that facilitate further scientific advances. This is commonly done in scientific articles where text is the main component. However, scholarly publications are evolving. Many journal publishers already adhere to the FAIR Principles and ask for metadata regarding data and software accompanying the text-based publication, including information about the availability, access conditions, licenses, etc. Data availability statements (DASs) are already required by many journals and strengthened to ensure data is deposited in FAIR-enabling repositories ( examples in FAIRsharing). Many journals have created data-centric articles, and many data-focused journals have been launched. These ensure that research digital objects, including data and software, are presented as first-class objects. Their creators are credited and encouraged to share them. Data availability in the FAIRest way possible has become essential to advancing research. Among other benefits, this allows for improving the reproducibility and replicability of research, having a positive effect on the trustworthiness and general quality of research, where making it FAIR means making it transparent. Journal publishers, therefore, will want to have access to trusted FAIRness assessment tools or outputs associated with the data to (i) assist and facilitate the peer review process; and (ii) create value filters/badges for the data articles. If such evaluations are not available, journal publishers may also want to (i) direct the authors to one or more tools powered by an approved set of FAIR metric tests recommended by the FAIR Metrics Governance body; or (ii) integrate the assessment into their existing internal submission system.

## 4. Stakeholder benefits from FAIRness governance

Table 1 summarises the stakeholders and how they could benefit from FAIRness governance, based on the groups and use cases described in Section 3.

**Table 1.** Stakeholders and examples of how they could benefit from FAIRness governance.

Group	Stakeholder	Examples of possible benefits
**GROUP A:** FAIR practitioners	Researchers and research- performing organisations (domain-agnostic)	Researchers, in general, will be able to: • Get advice from their communities on what FAIRness assessment tools adjust best to their needs • Compare and select platforms supporting FAIR metadata, if possible, tailored to their research community • Compare and choose platforms supporting FAIR metadata for their particular digital object
	Research Software Engineers	Software Engineers will be able to: • Obtain a FAIRness assessment from a tool recognised by the governance body together with a summary that they can publish along with their software and data
	Domain-specific Research communities	Research communities will be able to: • Get advice from the FAIRness governance body on what FAIRness assessment tools can be better adjusted or adapted to their own needs • Joining with FAIR researchers, provide metadata standards tailored to their community
**GROUP B:** FAIR custodians	FAIR support stewards & trainers	FAIR stewards will be able to: • Assess the FAIRness of external digital objects used by the researchers they support, e.g., using one of the tools applying the guidelines provided by the FAIRness governance body. • Use guidelines provided by the FAIRness governance to improve FAIRness from the digital object design phase • Estimate the FAIRness of digital objects as they advance in the different stages of development and tune any necessary adjustments to improve the final FAIRness
	FAIR Repositories	FAIR repositories will be able to: • Use guidelines and recommendations provided by the FAIRness governance to improve their practices • Show their FAIRness to stakeholders
	FAIR tools developers and operators	FAIR tools developers will be able to: • Assess the FAIRness of the tools they develop as they progress in the different development stages, with the objective of incremental improvement • Assess the FAIRness of the data they use in their software tools, knowing that the assessment is compatible no matter what FAIR evaluator tool they choose so, if needed, they can later move to another one
	FAIR researchers	FAIR researchers will be able to: • Define metadata standards that have been certified as FAIR so they can later be used by their communities in the creation of tools and data • Identify FAIR metadata standards that can be used as part of their standard • Provide guidelines to their community regarding the creation of metadata and standards that will assure their FAIRness once they are ready to be assessed
	FAIR certification bodies	FAIR Certification Bodies will be able to: • Become and demonstrate their FAIRness by following the guidelines provided by the FAIRness governance body. • Provide transparent information on how to get a certification with them that follows the guidelines of the FAIRness governance body.
**GROUP C:** FAIR decision and policymakers	Funding agencies	Funding agencies will be able to: • Identify trusted FAIR assessment tools that are assuredly within the scope of the FAIR Principles and provide a transparent assessment • Suggest community-based FAIR assessment tools recognised by the FAIRness governance body. (which, e.g., make sure that the tools are within the scope of the community) • Understand and compare FAIR assessments provided by, e.g., their preferred tool (which can be a generic one) and that one used by the funded parties (which can be a community-specific one)
	Governments	Governments will be able to: • Create clear and transparent policies on the FAIRness of digital objects generated using public funding
	Publishers	Publishers and journal editors will be able to: • Select a trusted general-purpose FAIR assessment tool and integrate it into the submission platform so the published digital objects get a FAIR "badge." • Define a research impact metric based on FAIRness by using an assessment tool that follows the FAIRness governance guidelines

## 5. Models for governance

Communities can operate under many different frameworks according to their own individualistic needs, which reflect the community's size, history, and mission. To support community-based initiatives that fall outside traditional grant-funded routes or are pre-competitive and broad, communities have followed several models: from loosely organised and time-limited working groups and informal grassroots initiatives to not-for-profit (NFP). While all options provide a group of collaborators, a means to work together to build a community effort that benefits from increased public identity (branding) and independence; the NFPs incorporated model also provides persistence and an ability to handle funds (e.g., from donations/sponsorship; registration for meetings; society memberships; journals and private or public grants) with limited personal liability. There are, however, also independent unincorporated communities, unincorporated groups that use fiscal sponsors, and incorporated groups. The models under which a community works in a legal and financial framework (the governance model) are part of a continuum of increasing complexity.

Different organisations within the Internet standards domain have governance models to provide practical approaches to how FAIRness assessments might be governed. The following subsections detail those with a solid international presence and a dedicated focus on the FAIR Principles and their practical application. The first subsection considers models from Web standards organisations: World Wide Consortium W3C and Internet Engineering Task Force. The second subsection focuses on international organisations focusing on the FAIR Principles and their practical application: European Open Science Cloud, FAIR Digital Objects Forum, GO FAIR Initiative, and Research Data Alliance Communities of Practice.

### 5.1. Models from web standards organisations

***5.1.1. Internet engineering task force.*** The Internet Engineering Task Force ( IETF) develops and promotes open standards through open processes. The mission of the IETF is to make the Internet work better by producing high quality, relevant technical documents that influence the way people design, use, and manage the Internet. Working Groups are the primary mechanism for developing IETF specifications and guidelines, many of which are intended to be standards or recommendations. The standardisation mechanism of the IETF is the RFC (Request For Comments), published as a Public Access Specifications (PAS). They become official documents of Internet specifications, communications, procedures, and events, validated by the community.

***5.1.2. W3C.*** The World Wide Consortium ( W3C) is an international community whose work revolves around standardising Web technologies. To accomplish this work, W3C follows processes that promote the development of high-quality standards based on the Membership, Team, and public consensus. W3C processes promote fairness, responsiveness, and progress in all facets of the W3C mission. Various W3C groups enable W3C to pursue its mission by creating Web standards, guidelines, and supporting materials. W3C is governed by its membership, providing transparent membership of individuals from all stakeholder groups, and a sustainable organisational model through membership fees. W3C develops technical specifications and guidelines (as *de facto* standards) through a process designed to maximise consensus about the content of a technical report, ensure the high technical and editorial quality, and earn endorsement by W3C and the broader community.

### 5.2. Models from organisations with a focus on data FAIRness

***5.2.1. EOSC.*** The European Open Science Cloud (EOSC) FAIR Metrics and Data Quality Task Force will implement the proposed FAIR metrics for EOSC by assessing their applicability across research communities and testing a range of tools to enable uptake. Recommendations will be made to update metrics and adopt tools as appropriate. In addition, the group will undertake state of the art to understand measures of data quality, conducting several case studies to identify standard features and dimensions to define an approach for EOSC.

***5.2.2. FDO Forum.*** The FAIR Digital Object (FDO) Forum aims to achieve better coherence amongst many initiatives working on FDO-based designs and implementations. FDO aims toward a new infrastructure, an architectural extension of the Internet – the Internet of FAIR Data and Services – that can help achieve an interoperable, stable, and persistent domain of digital artefacts and thus a reliable and usable digital memory for the coming centuries. The FDO Forum is proposed as a neutral place where policymakers, researchers, and technical experts can meet to exchange information about all relevant aspects related to FDOs. A sign-on process is being put in place.

***5.2.3. GO FAIR Initiative.*** The Global Open (GO) FAIR initiative ( GO-FAIR) derives its governance from both stakeholders, via the coordinators of the community-driven GO FAIR Implementation Networks (INs) who make up the ‘Stakeholders Forum’, and National/International Support Offices. IN Coordinators elect representatives to the Executive Board (EB), which additionally consists of members of the national and international support and coordination offices. Work priorities are primarily identified by the INs, and are communicated to the EB, who then functions to ensure that the support offices are capable of providing short and long-term support to those priorities, and finding synergies with other national and international initiatives. Scientific Advisory Boards may be convened when additional strategic guidance is required. GO FAIR is focused in three distinct areas: “GO BUILD” with a scope of technology; “GO CHANGE” with a scope of policy and incentives; and “GO TRAIN” with a scope of training and skills development. Aligned with the GO FAIR Initiative is the non-competitive, non-profit GO FAIR Foundation ( GFF), which is a key mechanism for the “professionalization” of all FAIR activities via, for example, advocacy for standards implementation, design and execution of formal certification processes, and engaging in public private partnerships that advance FAIR objectives.

***5.2.4. RDA communities of practice.*** In the Research Data Alliance ( RDA), Communities of Practice (CoP) form to build discipline or domain-specific communities within RDA and investigate, discuss and provide knowledge and skills on any specific discipline and research domain issues relevant to the community and RDA. CoPs are composed of experts from the community interested in the research domain/discipline and are committed to directly or indirectly enabling data sharing, exchange, and interoperability. CoPs serve as platforms for communication and coordination among individuals, bridging the community outside and within the RDA with shared interests.

These are only a few examples of how a FAIRness governance body could be organised. However, what is clear is that the impetus to establish such a body must come from the stakeholder communities themselves. With this in mind, we are proposing this whitepaper as an invitation to begin such a discussion – a discussion that we will now seed with our perspectives on what features are expected from a body claiming governance of FAIR assessment

### 5.3. Key considerations for governance of FAIR assessment

There are several features of FAIR assessment governance that make it distinct from many of the organizations mentioned above. For example:

1) After an initial bootstrapping phase to address the existing set of metrics and tools, the frequency of updates will be modest, thus it is pragmatic to consider that stakeholders will participate largely without financial remuneration due to the small time-investment involved.
2) The task of the governance body will be largely the same over time and for all cases, distinguishing it from all of the bodies above, that have working groups or communities of practice that address distinct tasks or standards.
3) The makeup of the assessment governance body for any given new request must include (at a minimum) a domain-expert who knows the domain standards and data semantics; a FAIR expert who understands the Principles and their intent; and a software developer who will ensure that the assessment tool is executing the assessment accurately. Thus these governance bodies are likely to be extremely fluid in their membership, and short-lived, being established for very specific tasks.

Despite point 3 above, we anticipate that there will need to be a core of trusted stakeholders – primarily representing the FAIR community (both public and private) – who will assemble appropriate membership teams for these short-term tasks. We suggest that, similar to the W3C governance, the membership of this core group is time-limited; however, members may only be selected and/or nominated by other similar experts, and perhaps might serve on a rotational basis, to ensure the required expertise remains in-place for effective governance.

It seems reasonable that the EOSC FAIR Metrics and Data Quality Task Force would act as both the seed organization to begin this stakeholder discussion, as well as put forward TF members as nominees for the inaugural governance body, to be voted-on by the self-assembled stakeholder community.

## 6. Closing thoughts

In 2016, the High-Level Expert Group advising the European Commission on the nascent EOSC made a firm recommendation regarding FAIR data publishing: "Horizon 2020, should only support projects that properly address Data Stewardship [and those] that do not specify FAIR conditions… *should not be eligible for funding*.” ( Realising the Open Science Cloud, p. 18). Since then, FAIR has figured with increasing prominence in Horizon 2020 instructions, including running a (voluntary) FAIR-focused data stewardship pilot for all Horizon 2020 funded research projects. Similar requirements are appearing in many other countries and organisations. This is, however, somewhat of a trap due to the lack of a FAIRness assessment governance body.

Researchers, seeing that their funding or their ability to publish may shortly be dependent on their adherence to the FAIR Principles, have little choice but to claim to be FAIR. In contrast, the funding agency or journal editor, in turn, has no way to validate those claims because no identifiable body exists from which a trusted, expert-vetted set of assessment tests could be recommended. The emergent cottage industry of FAIRness assessment tool creation generates products that produce strikingly different results, even for the same digital objects. Thus, FAIRness is "stuck" between an increasingly common research and publishing requirement yet still an unmeasurable set of ideals.

This paper recognises two main areas where FAIRness governance is and will be longer-term required. On the one hand, there is an immediate need to harmonise the interpretation and evaluation of existing maturity indicators and FAIRness assessment tools, in particular those from the Maturity Indicators ^10^ that arose from the RDA FAIR Maturity Model Working Group; on the other hand, the FAIR Principles - in particular, Principle R1.3: “(Meta)data meet domain-relevant community standards” (also tackled by the RDA FAIRsharing WG)- forecasts extension of maturity indicators in a community-specific manner over time. In addition, differences across digital objects and the need to adapt the original FAIR Principles, mostly tailored to data, have already been recognised by government bodies such as the European Commission ^11^. There are ongoing efforts to adapt the FAIR Principles to research software ^12^ ( joint working group hosted at RDA), computational workflows ^13^, machine learning ^14^ (and at events such as RDA plenaries 17 and 18), and Virtual Research Environments ( via a working group at RDA). This will require a degree of oversight in the longer term to ensure the new assessments stay true to the intent of the Principles and are correctly focused on relevant, measurable FAIR features.

The benefits of establishing such a governance body are highlighted by identifying ten distinct stakeholder communities in hypothetical use cases and exploring examples of how each could take advantage of a trusted and consistently applied set of metrics and tests. These communities cannot be appropriately served until the FAIRness assessment reaches an adequate level of professionalism. The argument is made that this, in turn, requires FAIRness governance.

The need for governance around Web technologies and standards is not unique to FAIR. It has been addressed by a wide range of internationally scoped organisations whose outputs need to be trusted by both academic and commercial stakeholders. A variety of these is discussed, examining their governance models and exploring their benefits and possible deficiencies concerning the task of governance of FAIRness assessments.

The current state of FAIRness assessment metrics and tools does not provide sufficient accuracy and reproducibility to be fit-for-purpose. A wide range of stakeholders will benefit from the emergence of a governance mechanism whereby existing. Future FAIRness metrics and metric tests can be assumed to be: a) adequately aligned with the intention of the FAIR Principles, b) well-designed and cognizant of appropriate standards while recognising differences between communities and disciplines. This document is presented as an invitation to FAIR stakeholders to initiate a focused discussion around the need for FAIRness governance. This grassroots group could begin to jointly design a trusted governance mechanism, broadly representative of all stakeholder communities, appropriately scoped, and self-sustaining. As an initial forum for these discussions, we have created an open Google Group (fair-assessment-governance@googlegroups.com; membership can be requested). This forum can be used to provide feedback on this whitepaper, explore additional options for a governance structure, and provide opinions regarding the adequacy of the scope of this initial framing of the needs. All readers are invited to participate in this conversation as individuals or as representatives of stakeholder groups.

## Ethics and consent

Ethical approval and consent were not required.

## Data Availability

No data are associated with this article.
