# Supplementary material for: Community-driven governance of FAIRness assessment: an open issue, an open discussion
Source: Open Res Eur. 2023 Sep 6;2:146. Originally published 2022 Dec 23. [Version 2] doi: 10.12688/openreseurope.15364.2 (PMC10828551; doi:10.12688/openreseurope.15364.2)
Supplement: Peer review history

# Reviewer report: Reviewer response for version 2

**DOI:** 10.21956/openreseurope.17923.r36887

**Published:** 2024-02-15

**Authors:** Sheeba Samuel

**Affiliations:** 1 University of Jena, Jena, Germany

The article aims to establish the need for a governance model centered around a standardized definition of FAIRness. The article discusses the challenges associated with implementing the assessment of  FAIR (Findable, Accessible, Interoperable, and Reusable) Data Principles. It highlights the lack of a conclusive method to determine the required level of FAIRness for research artifacts, leading to confusion among stakeholders. The FAIR Principles, while covering various digital objects, focus on reusability and provide principles rather than standards, resulting in varied interpretations. The whitepaper identifies three stakeholder categories (FAIR practitioners, custodians, and decision and policymakers) and provides the use cases where stakeholders can benefit from the FAIR assessment governance. The classification of stakeholders into three distinct groups provides a clear structure for understanding the diverse roles and responsibilities in the FAIR governance model. It also examines governance models from other organizations to gain insights into the requisite decision-making processes.

 The timeliness of FAIRness assessment governance is evident due to multiple interpretations of FAIR principles by different FAIR assessment metrics and tools. The article explicitly outlines the stakeholders and their respective roles in benefiting from FAIRness assessment governance.

 However, a number of questions arises on the implementation of FAIRness assessment governance.

1. Can the diversity in community-driven governance models for FAIRness assessments still lead to varying interpretations of FAIR principles and their assessment?
2. How can FAIRness governance ensure a standardized understanding and implementation of these principles across different communities?
3. How to address potential challenges or conflicts that might arise among different stakeholder groups, offering a more nuanced perspective on the practical implementation of the proposed governance model? How the FAIRness governance model can adapt to the evolving changes in the roles and responsibilities of stakeholders over time?
4. Though these questions are out of scope for this paper, I look forward to the discussions that the paper will generate on implementing the governance model for FAIRness assessment.

Where applicable, are recommendations and next steps explained clearly for others to follow? (Please consider whether others in the research community would be able to implement guidelines or recommendations and/or constructively engage in the debate)

Yes

Does the article adequately reference differing views and opinions?

Yes

Are all factual statements correct, and are statements and arguments made adequately supported by citations?

Yes

Is the rationale for the Open Letter provided in sufficient detail? (Please consider whether existing challenges in the field are outlined clearly and whether the purpose of the letter is explained)

Yes

Is the Open Letter written in accessible language? (Please consider whether all subject-specific terms, concepts and abbreviations are explained)

Yes

Reviewer Expertise:

My research focuses on reproducibility, FAIR principles, knowledge engineering, provenance, and research data management.

I confirm that I have read this submission and believe that I have an appropriate level of expertise to confirm that it is of an acceptable scientific standard.

# Reviewer report: Reviewer response for version 2

**DOI:** 10.21956/openreseurope.17923.r36884

**Published:** 2024-01-30

**Authors:** Luiz Gadelha

**Affiliations:** 1 German Cancer Research Center (DKFZ), Heidelberg, Germany

In this manuscript, the authors recognize that there are currently no widely accepted, systematic, and conclusive way of assessing research artifacts with respect to which extent they follow the FAIR principles. Models for FAIR assessment governance inspired by well-known standardization bodies and technical associations are presented and discussed. In the context of the FAIR principles they associate governance with methodologies, techniques, and tools agreed by the community for supporting the FAIR principles. Key objectives and indicators of success were identified: the objectiveness and transparency of evaluation, the understanding and trust in the assessment process, and the adaptability of FAIRness criteria to the requirements of different domains. 

 They identify three levels of stakeholders and provide examples of requirements for each level. There stakeholder groups are defined according to their role w.r.t. the FAIR principles: (A) practical application of FAIR Principles in research, (B) providing support and tools, and (C) decision-making and policy formulation without direct involvement in FAIRification. 

 Overall, the manuscript brings as an important contribution a proposal for the governance of FAIR Principles evaluations with identification of stakeholders and use cases that was very needed and will benefit the research data community considerably. Next, a few comments and suggestions are provided:

 In section 3.3.3 regarding creating value filters/badges for the data articles, it might be interesting to mention the ACM Artifact Reviewing and Badging (https://www.acm.org/publications/policies/artifact-review-badging) as an example from the reproducibility point of view.

 In section 5.3, the authors state that "the frequency of updates will be modest" and that "The task of the governance body will largely be the same over time for all use cases". However, it might be the case that with new communities joining, new requirements will be brought in or discovered and new properties will also be subject to testing/verification. Data management best practices include not only the FAIR principles, as evidenced by the Five Safes Framework. I believe that the activities and responsibilities of such governance entities will be open-ended and constantly evolving. In this direction, the authors state in the manuscript that an expansion of expected FAIRness behaviors over time is expected, as the FAIR principles are adapted / contextualized to different domains.

 Some abbreviations are not completely defined or no references/links provided like:

 FAIR-4-Plants on sec. 3.1.2

 GO FAIR Implementation Network on sec. 3.1.3

 EOSC FAIR Metrics and Data Quality Task Force 

 Minor typographic errors:

 Pg 5., fourth paragraph, sec. 5.2.3, and Sec. 6: whitepaper

 Sec. 3.1.3: edits, such as...

 Sec. 5.1.2: should be World Wide Web Consortium

Where applicable, are recommendations and next steps explained clearly for others to follow? (Please consider whether others in the research community would be able to implement guidelines or recommendations and/or constructively engage in the debate)

Yes

Does the article adequately reference differing views and opinions?

Yes

Are all factual statements correct, and are statements and arguments made adequately supported by citations?

Yes

Is the rationale for the Open Letter provided in sufficient detail? (Please consider whether existing challenges in the field are outlined clearly and whether the purpose of the letter is explained)

Yes

Is the Open Letter written in accessible language? (Please consider whether all subject-specific terms, concepts and abbreviations are explained)

Yes

Reviewer Expertise:

Scientific data management, scientific workflows, provenance, and high performance computing

I confirm that I have read this submission and believe that I have an appropriate level of expertise to confirm that it is of an acceptable scientific standard.

## References

[1] Artifact Review and Badging – Version 1.0 (not current). *Association for Computing Machinery* .2020; Reference source

# Reviewer report: Reviewer response for version 2

**DOI:** 10.21956/openreseurope.17923.r34940

**Published:** 2023-09-27

**Authors:** Mark Leggott

**Affiliations:** 1 International Relations, Digital Research Alliance of Canada, Ottawa, ON, Canada

I find that the authors have addressed the suggestions/concerns in my original review, and I look forward to following the discussion that the article will generate. 

 The word "scuach" needs to be corrected.

Where applicable, are recommendations and next steps explained clearly for others to follow? (Please consider whether others in the research community would be able to implement guidelines or recommendations and/or constructively engage in the debate)

Yes

Does the article adequately reference differing views and opinions?

Partly

Are all factual statements correct, and are statements and arguments made adequately supported by citations?

Yes

Is the rationale for the Open Letter provided in sufficient detail? (Please consider whether existing challenges in the field are outlined clearly and whether the purpose of the letter is explained)

Yes

Is the Open Letter written in accessible language? (Please consider whether all subject-specific terms, concepts and abbreviations are explained)

Yes

Reviewer Expertise:

I am a practitioner with a focus on: digital research infrastructure, FAIR Principles, research data management, community building, research software, governance.

I confirm that I have read this submission and believe that I have an appropriate level of expertise to confirm that it is of an acceptable scientific standard.

# Reviewer report: Reviewer response for version 1

**DOI:** 10.21956/openreseurope.16609.r30516

**Published:** 2023-03-14

**Authors:** Mark Leggott

**Affiliations:** 1 International Relations, Digital Research Alliance of Canada, Ottawa, ON, Canada

The article deals with an important gap in the research data ecosystem: realizing the full potential of the FAIR principles via open and robust mechanisms, including oversight. In particular the emphasis on providing "supportive advice" in response to a FAIR assessment seems particularly compelling, and more likely to see an uptake of FAIR approaches. The details regarding stakeholders and associated use cases are especially helpful. The point made in section 6. that " *Researchers, seeing that their funding or their ability to publish may shortly be dependent on their adherence to the FAIR Principles, have little choice but to claim to be FAIR.*" is quite true in my experience. The challenge is how to best move the proposed approach forward in a global context where FAIR is now a part of a very complex fabric.

 Some of the Use Cases do more to highlight the potential for a substantial bottleneck in individuals sending questions or requests to the "FAIRness governance body", than to illustrate the use case. This issue (the bottleneck) could be expanded on in 5.3 or another appropriate section. In fact this could be a 4th feature of such a body in section 5.3.

 There is no discussion of another option for a "FAIRness governance body", that of a looser but coordinated federated model of existing bodies and organizations, building on the strengths of each. This could be presented in section 5.2 or 5.3 as a possible model.

 I might also have expected to see mention of a "FAIRness governance body" that could be focused primarily on the stakeholders in section 3.2, which may present a more sustainable approach to achieving the laudable, but challenging, goal highlighted in the article. In other words, a train-the-trainer approach to achieving the goals.

 I found myself returning to the traditional model of peer review as I read this document, and how that contrasts with the development of a "FAIRness governance body". The traditional peer review approach, while arguably flawed in some respects, is clearly sustainable in an ecosystem with a high volume of material. The authors may want to consider contrasting their proposed approach with others like peer review, especially with respect to sustainability. Having said that, this letter does at least get the conversation started, so it is a very valuable and timely intervention in the landscape.

 I realize the value of grounding a discussion of the proposed approach with the EOSC FAIR Metrics and Data Quality Task Force, but I sensed a disconnect between the focus of this group (ie. including data quality, which the article does not discuss) and the intent of the proposed approach. It may come down to a practical decision about how best to move the conversation forward, and that generally points to an existing body or group, or assemblage of same.

 There are some venerable and effective governance models/organizations listed in Section 5: I'm not sure that the FDO Forum is a good fit in this list, as it itself is still wrestling with the "right" approach to governance.

 The use case for 3.1.3 seems a bit complex, and a bit more of an "inside baseball" example than seems appropriate for this document.

 Additional links or references could be provided to ensure the reader is able to understand the context. For example, mention of the GO FAIR Implementation Network in 3.1.3 could include a link/citation.

 I assume the article will undergo a copyedit? There are a substantial number of mistakes and errors that need to be corrected.

 In summary I believe this will be an important stage in the conversation of the "operationalization" of the FAIR principles, and is all suited to the function of a Letter. While I have suggested some areas for improvement, they would be easily addressed, and result in a nice addition to the global conversation.

Where applicable, are recommendations and next steps explained clearly for others to follow? (Please consider whether others in the research community would be able to implement guidelines or recommendations and/or constructively engage in the debate)

Yes

Does the article adequately reference differing views and opinions?

Partly

Are all factual statements correct, and are statements and arguments made adequately supported by citations?

Yes

Is the rationale for the Open Letter provided in sufficient detail? (Please consider whether existing challenges in the field are outlined clearly and whether the purpose of the letter is explained)

Yes

Is the Open Letter written in accessible language? (Please consider whether all subject-specific terms, concepts and abbreviations are explained)

Yes

Reviewer Expertise:

I am a practitioner with a focus on: digital research infrastructure, FAIR Principles, research data management, community building, research software, governance.

I confirm that I have read this submission and believe that I have an appropriate level of expertise to confirm that it is of an acceptable scientific standard, however I have significant reservations, as outlined above.

# Response

**Published:** 2023-09-04

**Authors:** Leyla Jael Castro

**Affiliations:** ZB MED Information Centre for Life Sciences, Germany

Community-driven governance of FAIRness assessment: an open issue, an open discussion We thank the reviewers for taking the time to go through our manuscript. The comments have helped us improve the paper. We present here answers to the questions raised by reviewers and how we addressed the comments in the revised version.

 Reviewer 2 The article deals with an important gap in the research data ecosystem: realizing the full potential of the FAIR principles via open and robust mechanisms, including oversight. In particular the emphasis on providing "supportive advice" in response to a FAIR assessment seems particularly compelling, and more likely to see an uptake of FAIR approaches. The details regarding stakeholders and associated use cases are especially helpful. The point made in section 6. that "Researchers, seeing that their funding or their ability to publish may shortly be dependent on their adherence to the FAIR Principles, have little choice but to claim to be FAIR." is quite true in my experience. The challenge is how to best move the proposed approach forward in a global context where FAIR is now a part of a very complex fabric. Some of the Use Cases do more to highlight the potential for a substantial bottleneck in individuals sending questions or requests to the "FAIRness governance body", than to illustrate the use case. This issue (the bottleneck) could be expanded on in 5.3 or another appropriate section. In fact this could be a 4th feature of such a body in section 5.3.

 Response We address this directly in Section 5.3 point #1.  The bottleneck (if it exists) will be for tool developers - general questions about FAIR and issues with implementing it will not come to this governance body, but will be managed by their own FAIR community.  Since FAIR testing began, in late 2016, only 25 tests have been created for one of the most popular automated testing services (the FAIR Evaluator), despite having a completely open GitHub-based test submission process.  As such, we do not expect many tests will be submitted, nor with a frequency to become a bottleneck.  Moreover, as described in Section 5.3, we anticipate that the governance body will be composed of short-lived working groups, with differing membership in each instance.  This should scale, even if the number of submissions is higher than expected.

 There is no discussion of another option for a "FAIRness governance body", that of a looser but coordinated federated model of existing bodies and organizations, building on the strengths of each. This could be presented in section 5.2 or 5.3 as a possible model.

 Response How the FAIRness governance body communicates and coordinates with other related organizations would be part of the activities of such a body. This is outside the scope of this paper. 

 I might also have expected to see mention of a "FAIRness governance body" that could be focused primarily on the stakeholders in section 3.2, which may present a more sustainable approach to achieving the laudable, but challenging, goal highlighted in the article. In other words, a train-the-trainer approach to achieving the goals.

 Response We agree that a train-the-trainer (i.e., stakeholders) would be a good approach to distributing the expertise that leads to good evaluation tool design; however we see this would be a role more for the FAIR communities and the FAIR custodians rather than the FAIRness Governance body.  There still needs to be an objective measure of success, by some trusted “authority”.  The sustainability of peer-review is demonstrable in the scholarly publishing community.

 I found myself returning to the traditional model of peer review as I read this document, and how that contrasts with the development of a "FAIRness governance body". The traditional peer review approach, while arguably flawed in some respects, is clearly sustainable in an ecosystem with a high volume of material. The authors may want to consider contrasting their proposed approach with others like peer review, especially with respect to sustainability. Having said that, this letter does at least get the conversation started, so it is a very valuable and timely intervention in the landscape.

 Response This is an excellent observation, and is very much aligned with how we had envisioned governance would work (though it is up to the seed group to make the final determination).  Text has been added to the end of Section 5.3 to compare our initial ideas with the well-established process of peer-review.

 I realize the value of grounding a discussion of the proposed approach with the EOSC FAIR Metrics and Data Quality Task Force, but I sensed a disconnect between the focus of this group (ie. including data quality, which the article does not discuss) and the intent of the proposed approach. It may come down to a practical decision about how best to move the conversation forward, and that generally points to an existing body or group, or assemblage of same.

 Response EOSC FAIR Metrics and Data Quality Task Force has 2 sub-teams: one focusing on FAIR Metrics and the other on Data Quality Task Force. This paper is the output of the first sub-group, and for information to this reviewer the second group has produced the following report: https://doi.org/10.5281/zenodo.7515816

 There are some venerable and effective governance models/organizations listed in Section 5: I'm not sure that the FDO Forum is a good fit in this list, as it itself is still wrestling with the "right" approach to governance.

 Response After exploration of the nascent governance mechanism, and discussion with participants, we agree that their governance mechanism is still too undeveloped to be listed here.  That paragraph has been removed. The reviewer raises a fair point. We have modified the text to reflect this.

 The use case for 3.1.3 seems a bit complex, and a bit more of an "inside baseball" example than seems appropriate for this document.

 Response This use-case has been simplified.

 Additional links or references could be provided to ensure the reader is able to understand the context. For example, mention of the GO FAIR Implementation Network in 3.1.3 could include a link/citation.

 Response Footnote pointing to https://www.go-fair.org/implementation-networks/ added to page 8

 I assume the article will undergo a copyedit? There are a substantial number of mistakes and errors that need to be corrected.

 Response We have identified typos and corrected them In summary I believe this will be an important stage in the conversation of the "operationalization" of the FAIR principles, and is all suited to the function of a Letter.

 While I have suggested some areas for improvement, they would be easily addressed, and result in a nice addition to the global conversation

# Reviewer report: Reviewer response for version 1

**DOI:** 10.21956/openreseurope.16609.r30663

**Published:** 2023-02-17

**Authors:** Françoise Genova

**Affiliations:** 1 Strasbourg astronomical Data Center (CDS), Centre national de la recherche scientifique (CNRS), Strasbourg, France

The paper develops the case for establishing a community-driven governance of FAIRness assessment. The paper is based on a whitepaper already vetted in the EOSC context, but this referee used the criteria for publication in Open Research Europe to prepare this report.

 The whitepaper was included in the workflow of the EOSC Task Force on FAIR Metrics and Data Quality and the EOSC Board reviewed the final revision before publication, which means that the authors likely already gathered a range of comments from people with different knowledge and profiles. It aims at being “ *a starting point to foster an open discussion around FAIRness governance and mechanisms*”, and an open Google Group was created to engage the conversation.

 The paper explains in details the rationale to establish this community-driven governance. Key stakeholder groups and their use cases are identified and described to support the proposal. It may be useful however to consider an additional use case for a specific kind of “FAIR practitioners”: some domain-specific research communities, including this reviewers’ one, astronomy, have been organising themselves for a long time at the international level to define data exchange standards which cover many aspects of FAIR taking into account the specific community requirements – well in line with the understanding that “ *the FAIR principles would need to be fine-tuned and interpreted for individual research communities.*” These standards are widely implemented by data and tool producers and used by the community. One can guess that these communities will be considered somehow in the governance process, but their needs and capacities to contribute are somehow different from the use case developed in 3.1.3, which describes a community starting to develop its FAIR standards. The proposed governance is aimed at improving the situation on FAIR as a whole and it would be interesting to understand how this kind of legacy aspects would be taken into account.

 A number of organisations are cited as possible models for governance in Section 5. The length and level of details provided for the different organisations in Section 5.2 should be aligned. Also the RDA could be cited by itself as a relevant community-driven organisation, mentioning also its Community of Practices.

 For the next steps, it would be useful to summarize in a few sentences the activities proposed for the governing body, to clarify what is proposed as a starting point for the discussions during the next step. The third paragraph of Section 6 discusses on the one hand the alignment of FAIR criteria and tools and services developed to measure and enable FAIRness, and on the other hand the overseeing of the developments linked to FAIR for other research objects – these developments are currently done in other contexts. In addition, Box 1 states that “ *communities will have a process in place to collect requirements from their members*”. Does this mean that the governance body will be the place where a discussion on FAIR in a specific context (domain specific or adapted to other digital objects) will be possible, since it seems that the governing body will be the place where requirements will be collected? Or will the governing body collect requirements (and then fine-tune FAIR?) for communities which do not have other vehicles to do it? One could also understand from the paper that the governing body would rather be a clearinghouse for FAIR activities mostly developed elsewhere, which is somehow different and would not require the same amount of resources. Also, the authors seem to be confident in the fact that the FAIRness evaluation tools can be aligned, which is a core and critical role of the governing body in the current landscape. Is there a need to explicitly foresee a feasibility study which would inform the discussion to come?

 It is important to insist on the fact that the process should be community driven, as stated for instance in the paper title, and to enable it. Fostering an open debate as proposed is the right path forward. It could be noted that it will be particularly useful to find ways to engage with a wide variety of research communities since their role in fine-tuning and interpreting FAIR is recognised and their organisations and data cultures can be very different. Their interaction with the “FAIR custodians” to build a common understanding of the role and processes of the proposed governance body during the discussion phase is critical for success. Another critical point which could be mentioned is internationalisation, since by essence the whitepaper is produced in the European context. Also, the paper states that “ *It seems reasonable that the EOSC FAIR Metrics and Data Quality Task Force would act as both the seed organization to begin this stakeholder discussion, as well as put forward TF members as nominees for the inaugural governance body, to be voted-on by the self-assembled stakeholder community.*” It would be useful to explain how the possible perception of conflict of interest will be mitigated.

 Finally, it is appropriately stated in Section 2 that “ *FAIRness must not be used or perceived as an instrument to judge and punish*”, but as explained in Section 6 there is in practice an increasing tendency towards FAIRness being a condition for funding, also following a recommendation of the initial High Level Expert Group on EOSC. It would be useful to remind readers in the concluding thoughts that FAIR is essential as a starting point to improve science practices towards more reusability and reproducibility, beyond any top-down requirements from governance or funders.

Additional comments:

*Section 1*

- In the first paragraph, there is a mention of “ *Different research funders in other countries*”, not sure about what “other” refers to.
- The recent creation of the Coalition for Advancing Research Assessment (CoARA, https://coara.eu/) could be mentioned in addition to Reference 5. It was launched on December 12 ^th^, 2022, just before the completion of the first version of this paper.

Where applicable, are recommendations and next steps explained clearly for others to follow? (Please consider whether others in the research community would be able to implement guidelines or recommendations and/or constructively engage in the debate)

Partly

Does the article adequately reference differing views and opinions?

Yes

Are all factual statements correct, and are statements and arguments made adequately supported by citations?

Yes

Is the rationale for the Open Letter provided in sufficient detail? (Please consider whether existing challenges in the field are outlined clearly and whether the purpose of the letter is explained)

Yes

Is the Open Letter written in accessible language? (Please consider whether all subject-specific terms, concepts and abbreviations are explained)

Yes

Reviewer Expertise:

Science data sharing. Astronomy.

I confirm that I have read this submission and believe that I have an appropriate level of expertise to confirm that it is of an acceptable scientific standard, however I have significant reservations, as outlined above.

# Response

**Published:** 2023-09-04

**Authors:** Leyla Jael Castro

**Affiliations:** ZB MED Information Centre for Life Sciences, Germany

Community-driven governance of FAIRness assessment: an open issue, an open discussion We thank the editor and reviewers for taking the time to go through our manuscript. The comments have helped us improve the paper. We present here answers to the questions raised by reviewers and how we addressed the comments in the revised version. 

 Reviewer 1 The paper develops the case for establishing a community-driven governance of FAIRness assessment. The paper is based on a whitepaper already vetted in the EOSC context, but this referee used the criteria for publication in Open Research Europe to prepare this report.

 The whitepaper was included in the workflow of the EOSC Task Force on FAIR Metrics and Data Quality and the EOSC Board reviewed the final revision before publication, which means that the authors likely already gathered a range of comments from people with different knowledge and profiles. It aims at being “a starting point to foster an open discussion around FAIRness governance and mechanisms”, and an open Google Group was created to engage the conversation.

 The paper explains in details the rationale to establish this community-driven governance. Key stakeholder groups and their use cases are identified and described to support the proposal. It may be useful however to consider an additional use case for a specific kind of “FAIR practitioners”: some domain-specific research communities, including this reviewers’ one, astronomy, have been organising themselves for a long time at the international level to define data exchange standards which cover many aspects of FAIR taking into account the specific community requirements – well in line with the understanding that “the FAIR principles would need to be fine-tuned and interpreted for individual research communities.” These standards are widely implemented by data and tool producers and used by the community. One can guess that these communities will be considered somehow in the governance process, but their needs and capacities to contribute are somehow different from the use case developed in 3.1.3, which describes a community starting to develop its FAIR standards. The proposed governance is aimed at improving the situation on FAIR as a whole and it would be interesting to understand how this kind of legacy aspects would be taken into account.

 Response to new FAIR practitioner “Domain-specific research communities with established shared standards” We address this in point #3 of section 5.3 “The makeup of the assessment governance body for any given new request must include (at a minimum) a domain-expert who knows the domain standards and data semantics;” This expert would be expected to bring-in their knowledge of the legacy standards and norms within a given community.

 A number of organisations are cited as possible models for governance in Section 5. The length and level of details provided for the different organisations in Section 5.2 should be aligned. Also the RDA could be cited by itself as a relevant community-driven organisation, mentioning also its Community of Practices.

 Response The RDA communities of practice are mentioned in 5.2.4. RDA communities of practice We have also modified the text in 5.2 subsections to improve the balance w.r.t. length and level of details.

 For the next steps, it would be useful to summarize in a few sentences the activities proposed for the governing body, to clarify what is proposed as a starting point for the discussions during the next step. The third paragraph of Section 6 discusses on the one hand the alignment of FAIR criteria and tools and services developed to measure and enable FAIRness, and on the other hand the overseeing of the developments linked to FAIR for other research objects – these developments are currently done in other contexts. In addition, Box 1 states that “communities will have a process in place to collect requirements from their members”. 

1. Does this mean that the governance body will be the place where a discussion on FAIR in a specific context (domain specific or adapted to other digital objects) will be possible, since it seems that the governing body will be the place where requirements will be collected? 
2. Or will the governing body collect requirements (and then fine-tune FAIR?) for communities which do not have other vehicles to do it? 
3. One could also understand from the paper that the governing body would rather be a clearinghouse for FAIR activities mostly developed elsewhere, which is somehow different and would not require the same amount of resources. 

Response to Governing Body activities Activities are not detailed on purpose, as defining its scope and responsibilities would be a task of the nascent governance body itself. Questions

1. Not necessarily
2. Not necessarily
3. Without pre-judging the outcomes of the governance body establishment, this is what we believe is the most likely result. Communities that are implementing FAIR in-practice will (and are) organizing themselves.  When a metric and its test have been designed, they would then come to the governance body for a peer-review process that will ensure a basic level of harmonization (especially between communities) and quality of the test, and therefore of the FAIRness evaluators that utilize that test. 

Additional text has been added to the end of Section 5.3 that hopefully clarifies how we envision the governance could work.

 Also, the authors seem to be confident in the fact that the FAIRness evaluation tools can be aligned, which is a core and critical role of the governing body in the current landscape. Is there a need to explicitly foresee a feasibility study which would inform the discussion to come?

 Response  FAIRness evaluation tools could be aligned if they use a common agreed basis (algorithm) for metadata harvesting and probing. The question is not whether they could (since this is technically a fact) but if they will - it is not a technical problem but a social one. The scope and purpose of the governance is not aligning the evaluation tools per se but to define a peer-review process for tests; evaluation tools that use these tests will then become naturally aligned.  The motivation for using tests that have passed the peer-review process will (hopefully) be high, since we anticipate that agencies and regulatory bodies will prefer tests that have passed peer-review. Additional text has been added to Box 1 to better reflect this separation of concerns.

 It is important to insist on the fact that the process should be community driven, as stated for instance in the paper title, and to enable it. Fostering an open debate as proposed is the right path forward. It could be noted that it will be particularly useful to find ways to engage with a wide variety of research communities since their role in fine-tuning and interpreting FAIR is recognised and their organisations and data cultures can be very different. Their interaction with the “FAIR custodians” to build a common understanding of the role and processes of the proposed governance body during the discussion phase is critical for success. 

 Response to interaction between community and FAIR custodians We agree with the reviewer. This should be the job of the FAIR custodians. There are already bodies that undertake these activities (e.g. the GO FAIR Implementation Networks and RDA Communities) where fine-tuning and interpreting FAIR for their domains. FAIR custodians are described in section 3, second bullet point corresponding to the “second group (B)”.

 Another critical point which could be mentioned is internationalisation, since by essence the whitepaper is produced in the European context. Also, the paper states that “It seems reasonable that the EOSC FAIR Metrics and Data Quality Task Force would act as both the seed organization to begin this stakeholder discussion, as well as put forward TF members as nominees for the inaugural governance body, to be voted-on by the self-assembled stakeholder community.” It would be useful to explain how the possible perception of conflict of interest will be mitigated.

 Response to internationalization and conflict of interests The EOSC FAIR Metrics and Quality Task Force and other co-authors of this whitepaper are nothing more than a seed group starting the discussion. Text has been added to Section 5.3 to clarify what we anticipate will be the role of the Task Force. The sentence “as well as put forward TF members as nominees for the inaugural governance body, to be voted-on by the self-assembled stakeholder community” has also been removed.

 Finally, it is appropriately stated in Section 2 that “FAIRness must not be used or perceived as an instrument to judge and punish”, but as explained in Section 6 there is in practice an increasing tendency towards FAIRness being a condition for funding, also following a recommendation of the initial High Level Expert Group on EOSC. It would be useful to remind readers in the concluding thoughts that FAIR is essential as a starting point to improve science practices towards more reusability and reproducibility, beyond any top-down requirements from governance or funders.

 Response  We agree, we have added text to the final paragraph on Page 18, see paragraph starting by “Perhaps the most substantial benefit of FAIRness governance [...]”.

 Additional comments: Section 1

- In the first paragraph, there is a mention of “Different research funders in other countries”, not sure about what “other” refers to.

Response Indeed, the sentence was not clear. It was changed to “Funders may [...]”

- The recent creation of the Coalition for Advancing Research Assessment (CoARA, https://coara.eu/) could be mentioned in addition to Reference 5. It was launched on December 12th, 2022, just before the completion of the first version of this paper.

Response A mention of the CoARA web page was added right after Reference 5 in the main text.
